# New clues to the nature of immunoglobulin G4-related disease: a retrospective Japanese multicenter study of baseline clinical features of 334 cases

**DOI:** 10.1186/s13075-017-1467-x

**Published:** 2017-12-01

**Authors:** Kazunori Yamada, Motohisa Yamamoto, Takako Saeki, Ichiro Mizushima, Shoko Matsui, Yuhei Fujisawa, Satoshi Hara, Hiroki Takahashi, Hideki Nomura, Shigeyuki Kawa, Mitsuhiro Kawano

**Affiliations:** 1 Division of Rheumatology, Kanazawa University Hospital, 13-1, Takara-machi, Kanazawa, Ishikawa 920-8640 Japan; 2 Department of Advanced Research in Community Medicine, Kanazawa University Graduate School of Medical Sciences, Kanazawa, Japan; 3 Department of Rheumatology and Clinical Immunology, Sapporo Medical University School of Medicine, Sapporo, Japan; 4 Department of Internal Medicine, Nagaoka Red Cross Hospital, Nagaoka, Japan; 5 Health Administration Center, University of Toyama, Toyama, Japan; 6 Division of General Medicine, Kanazawa University Hospital, Kanazawa, Japan; 7 Department of Internal Medicine, Matsumoto Dental University, Shiojiri, Japan

**Keywords:** IgG4-related disease, Clinical features, Hypocomplementemia

## Abstract

**Background:**

The aim was to further characterize immunoglobulin G4-related disease (IgG4-RD) by a large-scale multicenter study of its clinical and laboratory features conducted by multidisciplinary physicians of IgG4-RD in Japan.

**Methods:**

Various specialists retrospectively evaluated IgG4-RD patients diagnosed between 1996 and 2015 in five hospitals by analyzing their baseline clinical features, laboratory, imaging, and pathological test findings, and treatment.

**Results:**

Of the 334 patients listed, 205 were male and median age at diagnosis was 65 years. The mean number of organs involved was 3.2 at diagnosis. The most frequently affected organs were the salivary glands, followed by the lacrimal glands, lymph nodes, pancreas, retroperitoneum/periaorta, kidneys, and lungs. The mean serum level of IgG4 was 755 mg/dl, and more than 95% of patients had elevated serum IgG4 levels. The median serum level of C-reactive protein (CRP) was 0.1 mg/dl and the level was less than 1 mg/dl in 90% of patients. A total of 34.7% of patients had low serum levels of C3. Serum levels of C3 and non-IgG4 IgG, calculated as the total IgG minus IgG4, showed an inverse correlation in patients with kidney lesions, while serum IgG4 levels were not correlated with serum C3 levels. Corticosteroid was administered in 78.0% of patients, and was effective in all.

**Conclusions:**

The serum CRP level is generally low and the serum IgG4 level is elevated in most Japanese IgG4-RD patients, in contrast to western patients. These original findings suggest that these two parameters in IgG4-RD differ in some interesting ways from those hitherto reported in western populations. Additional studies, especially international comparative ones, are needed to elucidate the extent and significance of these differences between populations. Attention will also have to be paid to whether the existence of such differences requires consideration when devising international classification criteria.

**Electronic supplementary material:**

The online version of this article (doi:10.1186/s13075-017-1467-x) contains supplementary material, which is available to authorized users.

## Background

Immunoglobulin G4 (IgG4)-related disease (IgG4-RD), a widely recognized systemic inflammatory disorder [1–3], affects a broad range of organs with main features of elevated serum IgG4 levels, copious infiltration of IgG4-positive cells into the affected organs, and a characteristic fibrosis called storiform fibrosis [2, 4]. IgG4-RD affects a broad range of organs such as the pancreas, lacrimal glands, salivary glands, kidneys, lungs, retroperitoneum and/or periaorta, skin, and lymph nodes [3, 5–8]. Owing to its extremely diverse clinical picture, individual clinicians encounter a clinically distinct patient population depending on his/her specialty and geographic location, making it difficult to avoid institutional bias in a single-center experience. Recently, several larger cohort studies have sought to characterize the clinical characteristics of IgG4-RD of over 100 patients [9–11]. Although these reports documented the baseline clinical features of IgG4-RD, differences were seen in the frequency of the affected organs and some laboratory data possibly due to different inclusion criteria, authors’ specialty, study design, and racial biases. This prompted us to conduct a large-scale multicenter study with well-experienced physicians of IgG4-RD including rheumatologists, gastroenterologists, pulmonologists, and nephrologists to clarify the baseline clinical and laboratory features of IgG4-RD in a large-scale cohort of 334 IgG4-RD patients.

## Methods

### Summary of cohort

We retrospectively evaluated 334 patients with IgG4-RD who were diagnosed between 1996 and 2015 in Kanazawa University Hospital, Sapporo Medical University Hospital, Nagaoka Red Cross Hospital, University of Toyama Hospital, Shinshu University Hospital, and affiliated hospitals. Data related to patients’ baseline clinical features, laboratory findings, imaging tests, pathological tests, and treatments were derived from their medical records.

### Inclusion criteria

To minimize bias regarding the organs affected by IgG4-RD, the diagnosis of IgG4-RD was made by specialists of IgG4-RD in each institution, supported by the comprehensive diagnostic criteria (CDC) [12] and/or criteria of each organ including type 1 autoimmune pancreatitis, IgG4-related kidney disease, IgG4-related Mikulicz’s disease (IgG4-related sialadenitis and dacryoadenitis), and IgG4-related sclerosing cholangitis [6, 13–15]. The CDC consist of three parts including clinical features showing characteristic diffuse/localized swelling or masses in single or multiple organs, elevation of serum IgG4 concentrations (≥135 mg/dl), and histopathological findings including marked lymphoplasmacytic infiltration, fibrosis, and infiltration of IgG4-positive cells (IgG4^+^/IgG^+^ > 40% and > 10 IgG4^+^ plasma cells/HPF). Since the cutoff number of IgG4-positive plasma cells of the CDC is lower than those of the Consensus statement on the pathology of IgG4-RD [16], we made the diagnosis of IgG4-RD referring to the Consensus statement on the pathology by specialists who have treated many IgG4-RD patients and paid particular attention to any clinicopathologic correlations. The histopathological importance of tissue infiltrating IgG4-positive plasma cells (IgG4^+^ PC) in the diagnosis of AIP was reported in 2002, and was also confirmed in the diagnosis of Mikulicz disease (MD) in 2005, which is currently known as IgG4-related dacryoadenitis and sialadenitis. Therefore, after 2005, almost all patients in our study were diagnosed as having IgG4-RD with reference to the immunohistochemical findings of IgG4 staining, clinical and imaging features, and/or serum IgG4 levels. In contrast, 30 patients who were diagnosed as having AIP or MD before 2005 were also included in our study. Of these, four patients were diagnosed with AIP before 2002. Reevaluation of histopathological samples was performed in all four of these patients, and the diagnosis of IgG4-RD was confirmed. Similarly, 26 patients were diagnosed with IgG4-related dacryoadenitis and sialadenitis before 2005. Fifteen of these 26 patients were reevaluated histopathologically and the final diagnosis was confirmed. In the remaining 11 patients, eight had bilateral lachrymal and submandibular gland swelling with elevated serum IgG4 levels, with this combination highly specific for the diagnosis of IgG4-RD. The other three patients had only one set of bilateral lacrimal or bilateral submandibular gland swelling, and all of them had an elevated serum IgG4 level. These three patients were diagnosed with IgG4-RD with reference to the clinical and imaging features and elevation of serum IgG4 levels.

The numbers of patients with definite, probable, and possible IgG4-RD according to CDC and/or organ-specific criteria were 280 (83.8%), 49 (14.7%), and 4 (1.2%), respectively. One patient was diagnosed clinically with IgG4-RD (Table 1).

**Table 1:** Baseline clinical features of patients with immunoglobulin G4-related disease

Characteristic	Value
Mean age at diagnosis (years), mean ± SD (range)	63.8 ± 11.5 (25–91)
Sex (*n*), male/female	205 (61.4%)/129 (38.6%)
Mean follow-up period (years)	4.2 ± 3.3
Diabetes mellitus, *n* (%)	110/321 (34.3)
Autoimmune pancreatitis (–)	71/237 (30.0)
Autoimmune pancreatitis (+)	39/84 (46.2)
Affected organs, *n* (%)	
Salivary glands	242 (72.7)
Lacrimal glands	190 (57.1)
Lymph nodes	188 (56.5)
Pancreas	85 (25.5)
Retroperitoneum/periaorta	83 (24.9)
Kidney	79 (23.7)
Lung	78 (23.4)
Prostate	32 (9.6)
Bile duct	18 (5.4)
Skin	5 (1.5)
Thyroid glands	3 (0.9)
Mean number (range) of affected organs	3.2 (1–11)
Number (%) of affected organs	
1	38 (11.4)
2	86 (25.7)
3	84 (25.1)
4	75 (22.5)
5	25 (7.5)
6	18 (5.4)
7	3 (0.9)
8	2 (0.6)
9	2 (0.6)
10	0 (0.0)
11	1 (0.3)

*SD* standard deviation

### Clinical features

We retrospectively analyzed the medical records of all patients included in this study. We noted the serum levels of IgG, IgG4, IgE, C3, C4, CH50, and C-reactive protein (CRP), the affected organs, the diagnostic imaging tests including computed tomography (CT), magnetic resonance imaging (MRI), gallium (Ga) scintigraphy, and positron emission tomography (PET), and the prevalence of biopsy of the affected organs. Serum IgG4 levels were measured using the nephelometric assay in all patients in this study. In addition, as a new serological marker, we defined non-IgG4 IgG as total IgG minus IgG4. Non-IgG4 IgG substitutes for the sum of IgG1, IgG2, and IgG3, and an increase of non-IgG4 IgG seems to mean that the serum level of IgGs which can activate the complement pathway is increased. We also evaluated the prevalence of corticosteroid therapy, average initial and maintenance dose of corticosteroid, effectiveness of corticosteroid therapy, and recurrence rate. The improvement of the affected organs was decided as the changes in symptomatic, radiologic, serologic, or histologic features. In IgG4-related kidney disease (IgG4-RKD), improvement of renal function was also considered. The definition of recurrence was the reappearance or worsening of symptomatic, radiologic, serologic, or histologic features of IgG4-RD. In IgG4-RKD, a rapid increase in the serum level of creatinine, after careful exclusion of other renal diseases, was also considered as recurrence. Reelevation of the serum levels of IgG or IgG4 alone was not regarded as recurrence. Furthermore, we determined the prevalence of diabetes mellitus (DM) at diagnosis of IgG4-RD and malignancy both before and after diagnosis of IgG4-RD.

### Statistical analysis

Differences between groups were assessed by chi-square test for categorical variables, and by Mann–Whitney *U* test for linear variables. The analysis of the organs associated with hypocomplementemia was performed using logistic regression analysis, adjusted for age, sex, and presence/absence of DM. Correlation analysis was performed to confirm the relationship between hypocomplementemia and serum levels of IgG4 or non-IgG4 IgG. Explanatory factor analysis followed by Varimax rotation was used to examine the structure of organ coinvolvement. All statistical tests were performed using SPSS software (version 22). Significant differences were defined as *p* < 0.05.

## Results

### Patient demographics

Two hundred and five patients were male, and 129 were female (male 61.4%). The mean and median ages at diagnosis were 63.8 ± 11.5 and 65 years (range 25–91). All patients were Japanese. The average follow-up period was 4.2 years. The prevalence of diabetes mellitus (DM) was 34.4% and was significantly higher in those with rather than without AIP (46.2% vs 30.0%, *p* = 0.005) (Table 1). Sixty-seven malignancies appeared in 57 of 334 patients (17.1%) with IgG4-RD (Table 2). Two and six patients had three and two malignancies, respectively. The types of malignancy are presented in Additional file 1: Table S1. Lung cancer was the most frequent malignancy in patients with IgG4-RD, followed by gastric cancer, colon cancer, and prostate cancer. Lung, colon, prostate, and renal cancers were frequently seen both before and after the diagnosis of IgG4-RD, whereas gastric cancer and malignant lymphoma tended to appear after the diagnosis of IgG4-RD (Table 2).

**Table 2:** Type of malignancies

Type of malignancy	Before diagnosis of IgG4-RD	After diagnosis of IgG4-RD
Total	30	36
Time before or after diagnosis of IgG4-RD (years)	6.1	2.7
Lung cancer	6	6
Colon cancer	4	5
Renal cancer	4	1
Prostate cancer	3	4
Gastric cancer	2	5
Breast cancer	2	0
Malignant lymphoma	2	5
Thyroid cancer	2	2
Bladder cancer	2	1
Cancer of the ovary	1	0
Uterine body cancer	1	0
Urothelial cancer	1	0
Pancreatic cancer	0	1
Cervical cancer	0	1
Leukemia	0	1
Tongue cancer	0	1
Cancer of the throat	0	1
Adenoma sebaceum	0	1
Gastrointestinal stromal tumor	0	1

*IgG4-RD* immunoglobulin G4-related disease

### Affected organs

The mean number of organs involved was 3.2 (range 1–11). The most frequently affected organs were the salivary glands (SG) (72.7%) followed by the lacrimal glands (LG) (57.1%), lymph nodes (56.5%), pancreas (25.5%), retroperitoneum (RP)/periaorta (24.9%), kidney (23.7%), and lung (23.4%). Single organ involvement was seen in 38 of 334 patients (11.4%) (Table 1). After excluding lymph nodes, the ratio of single organ involvement was 18.9%. The other affected organs with a prevalence of more than 1% were the prostate (9.6%), bile duct (5.4%), and skin (1.5%). The RP/periaorta, lung, and kidney were more frequently affected in males than in females, and the reverse for the LG (see Additional file 1: Table S1). The factor analysis identified three factors, explaining 21.7% of the total variance: factor 1 explained 8.1%, affecting LG, SG, and lymph node involvement; factor 2 explained 7.0%, affecting RP/periaorta involvement; and factor 3 explained 6.6%, and affected kidney and lung involvement. These results indicated that organ involvement occurs rather randomly, although there might be some tendency of coinvolvement in the three different organ categories (see Additional file 2: Table S2).

### Laboratory data

Mean serum levels of IgG and IgG4 were 2403 ± 1204 and 755 ± 642 mg/dl, respectively. Elevation of serum levels of IgG4 defined as IgG4 ≥ 135 mg/dl were seen in 318 of 333 patients (95.5%). Mean serum level of IgE was 611 ± 1198 IU/ml, and elevated serum levels defined as IgE ≥ 250 IU/ml were seen in 158 of 309 patients (51.1%). Elevation of serum levels of CRP defined as CRP ≥ 0.3 mg/dl was seen in 90 of 328 patients (27.4%). Mean and median serum levels of CRP were 0.42 and 0.10 mg/dl, respectively. In 79.9% and 90.2% of the patients, the serum level of CRP was less than 0.5 and 1.0 mg/dl, respectively (Table 3).

**Table 3:** Laboratory findings

Test item	Value
IgG (mg/dl)	2403 ± 1204
IgG4 (mg/dl)	755 ± 642
Elevation of IgG4 (≥135)	318/333 (95.5%)
IgE (IU/ml)	611 ± 1198
Elevation of IgE (≥250)	158/309 (51.1)
CRP (mg/dl)	0.42 ± 0.94
CRP (mg/dl), median	0.10
CRP < 0.5 mg/dl	262/328 (79.9)
CRP < 1.0 mg/dl,	296/328 (90.2)
Elevation of CRP (≥0.3)	90/328 (27.4)
C3 (mg/dl)	94.6 ± 31.5
C3 < 86	103/297 (34.7)
C4 (mg/dl)	21.2 ± 23
C4 < 17	100/297 (33.7)
CH50 (U/ml)	41.1 ± 15.5
CH50 < 25	46/292 (15.8)

Data presented as mean **±** standard deviation or number/total (%), unless indicated otherwise

*CRP* C-reactive protein, *IgE* immunoglobulin E, *IgG4* immunoglobulin G4

**Fig. 1:**
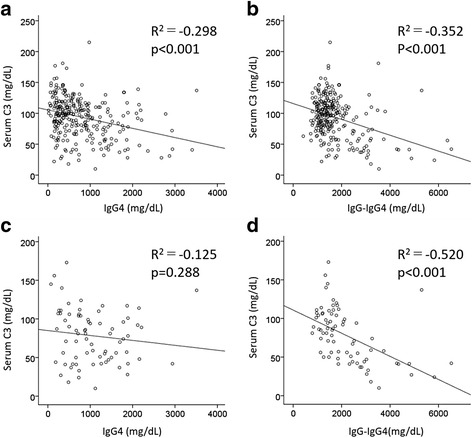
Pearson’s product-moment correlation coefficients for serum levels of C3 and IgG4 or other IgG subclass (IgG-IgG4). In the analysis of all patients, the serum level of C3 and that of both IgG4 and IgG-IgG4 showed inverse correlations (**a**, **b**). However, in the analysis of patients with kidney lesion, the serum level of C3 and serum level of IgG-IgG4 except for IgG4 showed inverse correlations (**c**, **d**)

Patients with low C3 and C4 defined as C3 < 86 mg/dl and C4 < 17 mg/dl were seen in 34.7% and 33.7%, respectively. The frequency of low C3 in patients with kidney lesion was significantly higher than in those without (55.4% vs 27.8%, *p* < 0.000) (Table 4). The frequency of low C3 in patients with pancreas or lung involvement was also significantly higher than in those without (pancreas 46.8% vs 30.3%, *p* = 0.009; lung 47.2% vs 30.7%, *p* = 0.015) (Table 4). We performed logistic regression analysis to explore the impact of organ involvement on hypocomplementemia, as well as that on severe hypocomplementemia. Kidney, lung, or pancreas involvement was independently related to hypocomplementemia, with odds ratios of 2.60 (95% CI 1.47–4.59, *p* = 0.001), 1.84 (95% CI 1.04–3.26), and 1.83 (95% CI 1.04–3.21), respectively (Table 5). Patients with severe hypocomplementemia defined as C3 < 50 mg/dl were seen in 30 of 297 patients (10.1%). In a logistic regression analysis, kidney or lung involvement, as well as DM, but not pancreas involvement, were strongly related to severely low C3, with odds ratios of 6.09 (95% CI 2.61–14.2), 2.47 (95% CI 1.05–5.50), and 2.38 (95% CI 1.01–5.50), respectively (Table 5). Next, we analyzed whether serum levels of IgG4 or non-IgG4 IgG, which is calculated as total IgG minus IgG4, were correlated with hypocomplementemia. In an analysis of all patients, the serum level of C3 was significantly inversely correlated with both serum levels of IgG4 and non-IgG4 IgG (Pearson’s product-moment correlation coefficient –0.298, *p* < 0.001 and –0.352, *p* < 0.001, respectively) (Fig. 1a, b), which indicated that low serum C3 level tended to occur in patients with high serum levels of both IgG4 and non-IgG4 IgG. On the other hand, in patients with kidney lesion, the serum C3 level was significantly inversely correlated with only serum levels of non-IgG4 IgG, which indicated that IgG4 may not affect the deposition of C3 in kidney tissue (Fig. 1c, d).

**Table 4:** Frequency of hypocomplementemia

	Frequency of hypocomplementemia		
Affected organ	Present	Absent	*p*
Kidney	41/74 (55.4%)	62/223 (27.8%)	<0.000
Pancreas	37/79 (46.8%)	66/218 (30.3%)	0.009
Lung	34/72 (47.2%)	69/225 (30.7%)	0.015
Lacrimal glands	52/162 (32.1%)	51/135 (37.8%)	0.329
Retroperitoneum/periaorta	30/76 (39.5%)	73/221 (33.0%)	0.330
Salivary glands	76/214 (35.5)	27/83 (32.5%)	0.685
Lymph nodes	61/171 (35.7%)	37/111 (33.3%)	0.703

Data presented as number/total (%)

**Table 5:** Factors related to hypocomplementemia

	Odds ratio	95% CI	*p*
Hypocomplementemia (C3 < 86)			
Kidney	2.602	1.474–4.593	0.001
Lung	1.844	1.044–3.256	0.035
Pancreas	1.830	1.044–3.209	0.035
Severe hypocomplementemia (C3 < 50)			
Kidney	6.091	2.607–14.229	<0.001
Lung	2.474	1.054–5.502	0.037
Diabetes mellitus	2.376	1.012–5.502	0.043

*CI* confidence interval

### Diagnostic tools

**Fig. 2:**
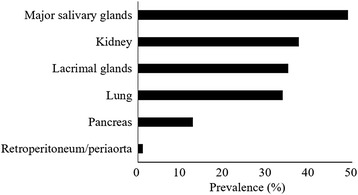
Type of affected organ biopsied. Major salivary glands were the most frequently biopsied organ, whereas the pancreas and retroperitoneum/periaorta were seldom biopsied

CT was performed in 99.4% of patients and contrast-enhanced CT in 93.1%. MRI, PET, and Ga scintigraphy were also performed in 30.2%, 45.5%, and 35.1% of patients, respectively, to determine the extent of systemic involvement. According to the histopathological evaluations, the most frequently biopsied organ were the SG (49.0%), followed by the kidney (37.5%), LG (35.1%), lung (33.8%), pancreas (12.8%), and RP/periaorta (1.2%) (Fig. 2). These data indicated that the major SG were the most easily accessible organ in patients with IgG4-RD. In contrast, only a single patient underwent RP/periaorta biopsy because these deeper structures are much more difficult to access.

### Treatment

Corticosteroid therapy was administered to 245 of 314 patients (78.0%). The mean initial and maintenance dose of prednisolone was 30.5 and 4.1 mg/day, respectively. Corticosteroid was effective in all patients. Recurrence was noted in 67 of 314 patients (21.3%), in whom 23 of 67 (34.3%) were not receiving corticosteroid at the time of recurrence. The average dose of corticosteroid at first recurrence was 7.1 mg/dl.

## Discussion

To the best of our knowledge, this is the largest cohort study thus far to collect IgG4-RD patients and analyze their clinical features. The salient findings of our cohort study can be summarized as follows. First, elevated serum levels of IgG4 were seen in more than 95% of patients with IgG4-RD. Second, the median serum CRP level was 0.1 mg/dl, and the serum CRP level was less than 1 mg/dl in 90.2% of the patients, meaning that serum levels of CRP are within the normal range in most IgG4-RD patients in Japan. Third, low C3 was seen in 34.7% of patients, and its frequency differed depending on the organs affected. The affected organs associated with low serum levels of C3 were the kidney, lung, and pancreas. Serum levels of C3 and non-IgG4 IgG calculated as total IgG minus IgG4 showed inverse correlations in patients with kidney lesion, while serum IgG4 levels did not correlate with serum C3 levels. Fourth, the most frequently affected organs were the salivary glands, lacrimal glands, pancreas, retroperitoneum/periaorta, kidneys, and lungs.

Major differences have been noted in the prevalence of affected organs in previously published studies [9–11]. Wallace et al. [9] analyzed IgG4-RD patients all of whom were subjected to pathological analysis. In their study, the prevalence of organs that were difficult to access, such as the pancreas, was relatively low and 19.2% of patients had type I AIP. Inoue at al. [10] used a radiology database to detect IgG4-RD patients who were pathologically diagnosed with IgG4-RD and/or fulfilled the diagnostic criteria of AIP. This method picked up AIP patients without pathological analysis and showed that 61% of patients had pancreas lesions. However, the prevalence of retroperitoneal and/or periaortal lesions was very low (both 4%) in both analyses, and the issue of how to best diagnose involvement of organs which are difficult to access remained unresolved. In our study, patients with retroperitoneal and/or periaortal lesions were diagnosed as having IgG4-RD without biopsy through clinicoimaging correlations obtainable in daily medical practice. We found six organs to be the most frequently affected: salivary glands, lacrimal glands, pancreas, retroperitoneum/periaorta, kidney, and lung, with the last four organs having almost the same prevalence (around 25%). The major affected organs could be classified into the following three subgroups using Varimax rotation: group 1, lacrimal glands, salivary glands, and lymph node; group 2, retroperitoneum/periaorta; and group 3, kidney and lung (see Additional file 2). Inoue et al. [10] reported that male and female patients showed a different distribution of organ manifestations. They noted that dacryoadenitis and sialadenitis developed more commonly in females (female-to-male ratio 41:18 and 57:29 respectively), while periaortitis was significantly more common in males than females (female-to-male ratio 2:25). Thus, our results of factor analysis are consistent with their results. Although we are not yet able to clarify the factors particular to each subgroup, recognition of these subgroups provides a good opportunity to speculate on the different pathogenetic mechanisms at work in each of them.

In this study, the serum IgG4 level was elevated in 95% of patients. Inoue et al. [10] analyzed 235 patients and found elevated serum IgG4 levels in 88% of them. Similarly, a large Chinese cohort study [11] showed that 97.5% of patients had serum IgG4 elevation. The results of these studies suggest that most patients with IgG4-RD in Asian countries have increased serum IgG4 levels (Table 6). In contrast, Wallace et al. [9] analyzed 125 patients with IgG4-RD and showed that only 51% of them had elevated serum IgG4 concentrations. In this study, 76% were Caucasian and only 8.8% were Asian. Therefore, the difference in the proportion of patients with elevated serum IgG4 in these studies may be due at least in part to racial differences. Another important factor possibly influencing the frequency of patients with high serum IgG4 is the proportion of patients with single organ involvement in each study. In the study by Wallace et al. [9] 38% of patients had single organ involvement, in contrast to 11% in our study. Similarly, only 4.2% had one organ involved in Lin et al.’s study [11] (Table 6). Since patients with single organ involvement tend to have low serum IgG4 levels, the percentage of such patients is thought to influence the frequency of high serum IgG4 patients.

**Table 6:** Comparison of laboratory findings and percentage of cases with single organ involvement between the present study and previous reports

	Present study	Inoue et al. [10]	Lin et al. [11]	Campochiaro et al. [22]	Ebbo et al. [23]	Fernández-Codina et al. [24]	Wallace et al. [9]
Reported year		2015	2015	2015	2012	2015	2015
Number of patients	334	235	118	41	25	55	125
Country	Japan	Japan	China	Italy	France	Spain	USA
Mean serum IgG level (mg/dl)	2403	NA	2300	NA	2550	NA	1573^d^, 1130^e^
Mean serum IgG4 level (mg/dl)	755	470^a^	1522	284^a^	720	223^a,b^, 93^a,c^	379^d^, 51^e^
Elevation of IgG4 (%)	95.5	88	97.5	73	100	NA	51.4
Median serum CRP level (mg/dl)	0.10	NA	NA	0.8	NA	NA	0.55^d^, 0.65^e^
Mean serum CRP level (mg/dl)	0.42	NA	0.66	NA	3.09	NA	NA
CRP < 0.5 mg/dl (%)	79.9	NA	NA	NA	NA	NA	NA
CRP < 1.0 mg/dl (%)	90.2	NA	NA	NA	46	NA	NA
Elevation of CRP (%)	27.4	NA	44.1	63	56	NA	67^d,f^, 46^e,f^
Single organ involvement (%)	11.4	42	4.2	58.5	12.0	52.7	38

*CRP* C-reactive protein, *IgG4* immunoglobulin G4, *IgG4-RD* immunoglobulin G4-related disease, *NA* not applicable

^a^Median value

^b^Highly suggestive IgG4-RD

^c^Probable IgG4-RD

^d^Patients with elevated serum IgG4 concentration

^e^Patients with normal serum IgG4 concentration

^f^Erythrocyte sedimentation rate and/or CRP

In our study, elevation of the serum level of CRP was seen only in 27.4% of patients. The median serum level of CRP was 0.1 mg/dl, and values less than 0.5 and 1.0 mg/dl were present in about 80% and 90% of IgG4-RD patients, respectively. These data indicate that serum levels of CRP in most patients with IgG4-RD are normal or low. Elevated serum IgG4 and pathological features mimicking those of IgG4-RD have been noted in other diseases such as multicentric Castleman disease (MCD) [17] and anti-neutrophil cytoplasmic antibody (ANCA)-associated vasculitis, and in particular granulomatosis with polyangiitis (GPA) [18] and eosinophilic granulomatosis with polyangiitis (EGPA) [19, 20]. Important for the differential diagnosis is the fact that serum CRP levels are generally elevated in these diseases [21], making the serum CRP level an important serological marker in differentiating IgG4-RD in Japanese patients. However, the fact that 44–67% of patients had elevated CRP levels in cohort studies from western countries [9, 22–24] indicates that interpretation of the serum CRP level may have to take into account racial and possibly other differences in the subjects and populations studied (Table 6).

Hypocomplementemia is an important serological feature of IgG4-RD, in particular IgG4-RKD [6, 25, 26]. More than 50% of IgG4-RKD patients have hypocomplementemia, while the prevalence of hypocomplementemia is about 30% in all IgG4-RD. This has led to speculation about the reasons underlying the strong association noted between hypocomplementemia and IgG4-related tubulointerstitial nephritis (TIN). In the present study, we noted low C3 and C4 levels in 35% and 34%, respectively, of IgG4-RD patients. Furthermore, hypocomplementemia in patients with kidney lesion was significantly more frequent than in those without, supporting previous reports.

Interestingly, serum complement levels rapidly normalize after successful corticosteroid therapy in parallel with the reduction of the size of mass or enlarged lesions [26]. Moreover, a renewed decrease in serum complement levels has been observed during the clinical course of relapse in patients with IgG4-related TIN [27], highlighting hypocomplementemia as a convenient biomarker of disease activity. However, in general, IgG4 has been thought not to bind to C1q, which is related to its inability to activate the classical complement pathway. On the other hand, one report showed that IgG4 molecules derived from patients with IgG4-RD and hypocomplementemia can activate complement through C1q-binding [28], although additional studies to confirm this are necessary. Previous histopathological immunofluorescent analyses showed that not only IgG4 but also IgG1 is deposited in the tubular basement membrane of the kidney. This suggests that IgG1 also has a pathogenetic role in IgG4-RKD. Using a mouse model of AIP, Shiokawa et al. [29] showed that more destructive injury in the pancreas was induced by injecting the patient with IgG1 rather than IgG4. In the present study, we found that serum levels of non-IgG4 IgG were significantly inversely correlated with serum C3 levels in patients with kidney lesion, while IgG4 itself has only a weak correlation with serum C3 levels. Our finding seems to be compatible with Shiokawa et al.’s result. To the best of our knowledge, the present study is the first report to show that non-IgG4 IgG subclasses exert a more significant influence on hypocomplementemia, probably through activation of complement in patients with kidney lesions, than IgG4 subclass. Thus, IgG subclasses other than IgG4 should also be focused on as pathogenetic immunoglobulins.

The rate of malignancies in this study (17.1%) was higher than those in other Japanese cohort studies (6.4% [10], 10.4% [30], 13.9% [31], 12.3% [32]) but lower than that of the study reported by Asano et al. (21.5% [33]). Of these five studies, three confirmed an increased risk of malignancy in IgG4-RD using the standardized incidence ratio (SIR), while the other two did not. Since we did not calculate the SIR because of the limitation of our study design, we could not easily compare the frequency of malignancy with that in other Japanese studies. The discordant results found in Japanese studies are likely attributable to methodological issues related to the study design of individual studies. Hirano et al. [32] excluded patients in whom malignancy was diagnosed ≤ 6 months before or after the onset of IgG4-RD to avoid selection bias, whereas other studies did not. Thus, careful evaluation is needed to conclude that the rate of malignancies in IgG4-RD is significantly higher than that of controls.

The recurrence rate of our study was lower than those of western countries. Reports from Spain [24] and Italy [22] described the recurrence rate to be 38.5% and 46%, respectively. On the other hand, Inoue et al. [10] noted the recurrence rate in Japanese patients to be 24%, almost the same as in our study. A recent meta-analysis [34] also showed that the recurrence rates in Americans and Europeans were higher than those in Asians. The difference in the recurrence rates between Japanese and Caucasians may be attributable to differences in the therapeutic strategy, especially the use or nonuse of corticosteroid maintenance therapy, race, and various other patient background features. However, we did not analyze the relationship between the treatment strategy for IgG4-RD such as the duration and tapering pace of corticosteroid and immunosuppressant administration and the recurrence rate. This important issue will require further studies for clarification.

Our study has several limitations. First, biopsies of the affected organs were not performed for 18.9% of the patients because the affected organs were not easily accessible. Second, methodological differences in the measurement of laboratory parameters such as serum complement levels among different periods and different institutions is a weak, but unavoidable, aspect of our retrospective cohort study. However, the effect of the difference of the method employed among different periods and different institutions on complement levels is limited and the influence would seem to be quite small. On the other hand, the serum IgG4 level was measured using nephelometry in all institutions. Therefore, we think no methodological difference existed. Third, we used the serum levels of non-IgG4 IgG instead of those of IgG1, IgG2, or IgG3, because we do not measure serum levels of IgG1, IgG2, and IgG3 in daily clinical practice.

It remains difficult to clarify the frequency of individual organs affected by IgG4-RD due to various biases related to the criteria employed for diagnosis and differences in the distribution of the specialties of clinicians making the diagnosis of IgG4-RD. In this study, we believe that we were better able to elucidate the baseline clinical features of IgG4-RD by having specialists of IgG4-RD from a variety of fields participate in the evaluations.

Another issue highlighted by this study is the finding of differences in various clinical parameters between our population and western ones. This raises the important question of whether the hitherto proposed international consensus criteria are equally applicable to all populations, and whether the more or less universally recognized clinical spectrum of IgG4-RD is the same in all of them. Further exploration of the reasons underlying any differences may also provide intriguing clues to the underlying nature of IgG4-RD and be of use in devising more accurate diagnostic criteria. To best characterize IgG4-RD and assess the universality of various criteria for diagnosis, future studies, including international comparative ones, will be needed with larger numbers of IgG4-RD patients diagnosed according to various criteria.

## Conclusions

In Japanese individuals, various clinical parameters in IgG4-RD including serum IgG4 and CRP levels appear to differ in some interesting ways from those hitherto reported in western populations. Additional studies, especially international comparative ones, will be needed to elucidate the extent and significance of these differences between populations. Attention will also have to be paid to whether such differences require consideration when devising diagnostic criteria that are meant to be applied internationally.

## Additional files

Additional file 1: Table S1. Gender differences in affected organs. (DOC 64.5 kb)

Additional file 2: Table S2. Factor analysis of the affected organs. (DOC 33 kb)

## Abbreviations

CDC: Comprehensive diagnostic criteria
CT: Computed tomography
DM: Diabetes mellitus
EGPA: Eosinophilic granulomatosis with polyangiitis
Ga: Gallium
IgG4: Immunoglobulin G4
IgG4-RD: Immunoglobulin G4-related disease
IgG4-RKD: Immunoglobulin G4-related kidney disease
LG: Lacrimal glands
MCD: Multicentric Castleman disease
MRI: Magnetic resonance imaging
PET: Positron emission tomography
RP: Retroperitoneum
SG: Salivary glands
TIN: Tubulointerstitial nephritis
