# Supplementary material for: New clues to the nature of immunoglobulin G4-related disease: a retrospective Japanese multicenter study of baseline clinical features of 334 cases
Source: Arthritis Res Ther. 2017 Dec 1;19:262. doi: 10.1186/s13075-017-1467-x (PMC5709928; doi:10.1186/s13075-017-1467-x)

**Additional file 1: Supplementary Table 1. Gender differences in affected organs.**

The numbers of patients with each affected organ. Retroperitoneum/periaorta, lung and kidney were more frequently affected in males than in females (p<0.001, p=0.001 and p=0.002, respectively), whereas lacrimal gland was more frequently affected in females than in males (p<0.001).


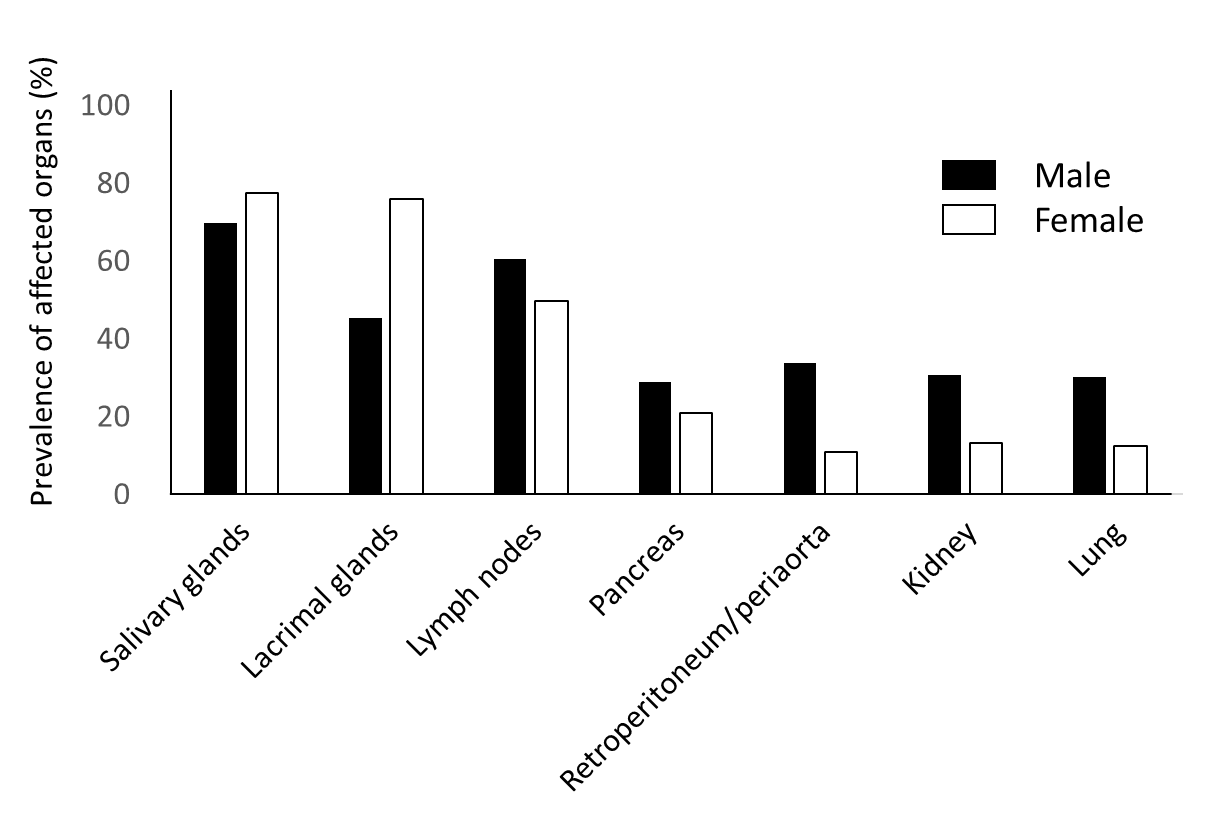

Supplement: Supplementary file 1 — Gender differences in affected organs. (DOC 64.5 kb) [file 13075_2017_1467_MOESM1_ESM.doc]
