# Supplementary material for: New clues to the nature of immunoglobulin G4-related disease: a retrospective Japanese multicenter study of baseline clinical features of 334 cases
Source: Arthritis Res Ther. 2017 Dec 1;19:262. doi: 10.1186/s13075-017-1467-x (PMC5709928; doi:10.1186/s13075-017-1467-x)
Supplement: Supplementary file 2 — Factor analysis of the affected organs. (DOC 33 kb) [file 13075_2017_1467_MOESM2_ESM.doc]

**Additional file 2: Supplementary Table 2. Factor analysis of the affected organs**

Factor 1 2 3

Pancreas -0.042 0.21 0.228

Lacrimal glands 0.340 -0.136 -0.355

Salivary glands 0.394 -0.086 -0.068

Kidney 0.127 0.179 0.324

RP/periaorta -0.002 0.598 0.029

Lung -0.044 -0.048 0.405

Lymph node 0.528 0.156 0.103
